# The VgrG Proteins Are “à la Carte” Delivery Systems for Bacterial Type VI Effectors*

**DOI:** 10.1074/jbc.M114.563429

**Published:** 2014-05-02

**Authors:** Abderrahman Hachani, Luke P. Allsopp, Yewande Oduko, Alain Filloux

**Affiliations:** From the MRC Centre for Molecular Bacteriology and Infection, Department of Life Sciences, Imperial College London, London SW7 2AZ, United Kingdom

**Keywords:** Bacterial Genetics, Bacterial Toxin, Microbiology, Protein Translocation, Pseudomonas aeruginosa (P. aeruginosa), Bacterial Cell Envelope, Type VI Secretion System, VgrG

## Abstract

The bacterial type VI secretion system (T6SS) is a supra-molecular complex akin to bacteriophage tails, with VgrG proteins acting as a puncturing device. The *Pseudomonas aeruginosa* H1-T6SS has been extensively characterized. It is involved in bacterial killing and in the delivery of three toxins, Tse1–3. Here, we demonstrate the independent contribution of the three H1-T6SS co-regulated *vgrG* genes, *vgrG1abc*, to bacterial killing. A putative toxin is encoded in the vicinity of each *vgrG* gene, supporting the concept of specific VgrG/toxin couples. In this respect, VgrG1c is involved in the delivery of an Rhs protein, RhsP1. The RhsP1 C terminus carries a toxic activity, from which the producing bacterium is protected by a cognate immunity. Similarly, VgrG1a-dependent toxicity is associated with the PA0093 gene encoding a two-domain protein with a putative toxin domain (Toxin_61) at the C terminus. Finally, VgrG1b-dependent killing is detectable upon complementation of a triple *vgrG1abc* mutant. The VgrG1b-dependent killing is mediated by PA0099, which presents the characteristics of the superfamily nuclease 2 toxin members. Overall, these data develop the concept that VgrGs are indispensable components for the specific delivery of effectors. Several additional *vgrG* genes are encoded on the *P. aeruginosa* genome and are not linked genetically to other T6SS genes. A closer inspection of these clusters reveals that they also encode putative toxins. Overall, these associations further support the notion of an original form of secretion system, in which VgrG acts as the carrier.

## Teaser

**Background:** The type VI secretion system (T6SS) is involved in bacterial warfare.

**Results:** Novel H1-T6SS-dependent toxins from *Pseudomonas aeruginosa* are identified using an *in vitro* bacterial killing assay.

**Conclusion:** The newly identified toxins showed a requirement for specific VgrG proteins.

**Significance:** VgrG proteins may represent an independent form of T6SS-dependent secretion, resembling the contact-dependent growth inhibition (CDI) system.

## Introduction

The type VI secretion system (T6SS)^4^ is present in most Gram-negative bacteria and is used to outcompete other microorganisms (1, 2). This machinery is mechanistically akin to the bacteriophage tail-like injection device (3), but the tail tube is composed of the bacterial Hcp protein (4). The puncturing device capping the Hcp tube has been identified as a member of the VgrG family of proteins. These proteins contain two conserved domains that share similarity with T4 phage bacteriophage proteins, namely the gp27 and the gp5 proteins, corresponding to the N- and C-terminal parts of VgrG, respectively (5). The gp5-like domain displays a rigid β-stranded helical structure and is shaped as a needle-like component. In a few cases it has been shown that the VgrG C terminus, which is considered as a spike, could further be sharpened by the addition of a protein carrying a PAAR domain, which adopts a rigid conical shape (6). The Hcp tube has been proposed to fit within a contractile sheath-like structure, which is composed of the TssB and TssC proteins (7–10). The contraction of the sheath results in the propelling of the tube and puncturing device across cell membranes (11–13). After contraction, the ClpV AAA ATPase disassembles the sheath components by binding the TssC proteins, and a new cycle of T6SS assembly can be restarted (12, 14).

In *Pseudomonas aeruginosa*, one of the three known T6SSs, H1-T6SS, is well characterized and is an archetypical example of a bacterial toxin delivery machine (4). Expression of the H1-T6SS cluster is regulated by a signaling cascade which involves the Gac/Rsm pathway (15–17). The RetS sensor counteracts the GacS sensor kinase (18). Thus, in a *P. aeruginosa retS* mutant, the Gac pathway remains active and leads to a constitutively active and functional H1-T6SS. All core components of the T6SS are encoded by the H1-T6SS cluster, which also contains a few additional accessory genes (19–21). Two *vgrG* genes, namely *vgrG1a* and *vgrG1b*, belong to this cluster (22). Although located on a different region of the chromosome, an additional gene, *vgrG1c*, is co-regulated with the H1-T6SS genes and thus induced in a *retS* background (22). Importantly, three gene couples have also been shown to be controlled by the RetS signaling pathways and encode toxin/immunity pairs involved in H1-T6SS-dependent bacterial killing (23–25). Tse1 and Tse3 have been characterized biochemically as amidases and are involved in the degradation of peptidoglycan. Tsi1 and Tsi3 are periplasmic immunity proteins and protect the cells from the deleterious effects of the cognate toxins. This family of toxins is broadly conserved in other T6SS-positive organisms such as *Serratia marcescens* (26, 27). The function of Tse2 remains elusive but is likely targeted to the cytoplasm where it exerts a bacteriostatic activity that could be counteracted by the Tsi2 protein (23). All three toxins are injected into neighboring cells, competitors, or siblings in an H1-T6SS-dependent manner. These toxins are very potent and allow *P. aeruginosa* to outcompete a broad range of other Gram-negative bacteria (28). Interestingly, whereas a *retS* mutant randomly attacks neighboring cells, a wild-type strain might only respond via a Tit-for-Tat mechanism (29).

Although the mechanism of assembly/contraction of the T6SS is beginning to be well documented (12), the precise mechanisms by which the toxins are delivered into the target cells remain elusive. The Hcp tube displays an internal diameter of 40 Å and could very well accommodate transiting unfolded effectors (4, 30). Recent data have demonstrated the presence of Tse2 protein within a hexameric Hcp ring (31). It was thus proposed that Hcp could act as a chaperone for T6SS effectors and not only as a component of the tail tube. These two functions are not exclusive as a stack of Hcp rings containing Tse2 could be fitted within the T6SS sheath, thus forming a pile of rings (or tube) that could be propelled by the contraction of the sheath. An alternative hypothesis came from the description of VgrG proteins displaying C-terminal extension with a catalytic activity, as exemplified by the VgrG3 protein of *Vibrio cholerae* (32, 33). This protein possesses a domain extension to its gp5 region that bears a peptidoglycan hydrolase activity. In this case the VgrG protein can be considered as the carrier located at the tip of the T6SS. Based on this observation, it is also reasonable to propose that genuine T6SS effectors could go for a “piggyback ride” by interacting with the tip of non-evolved VgrGs. A sophisticated concept has been proposed in which adaptors such as the PAAR proteins connect the T6SS toxin/effector to the tip of the VgrG proteins (6).

In the present study we lend support to this hypothesis by showing that the three VgrG proteins co-expressed with the H1-T6SS individually contribute to the toxicity exerted by a *retS* strain against *Escherichia coli* targets (34). This toxicity is observable in a background devoid of the characterized Tse1–3 toxins, thus revealing a broader repertoire of T6SS toxins.

## EXPERIMENTAL PROCEDURES

### 

#### 

##### Bacterial Strains and Growth Conditions

Bacterial strains used in this study are described in Table 1. *P. aeruginosa* strains were grown in Tryptone soy broth or LB supplemented with antibiotics where appropriate (streptomycin 2000 μg/ml, tetracycline 200 μg/ml) at 37 °C with agitation. *E. coli* strains were grown in LB broth supplemented with antibiotics where appropriate (streptomycin, 50 μg/ml; ampicillin, 50–100 μg/ml; kanamycin, 50 μg/ml; chloramphenicol, 34 μg/ml; tetracycline, 15 μg/ml).

**TABLE 1:** **Strains and plasmids used in this work**

Strain or plasmid	Relevant characteristics*^a^*	Source/Reference
**Strain**		
*E. coli*		
Dh5α	F−Φ80*lac*ZΔM15Δ (*lac*ZYA-*arg*F) U169 *rec*A1 *end*A1 *hsd*R17 (rK−, mK+) *pho*A *sup*E44 λ-*thi*-1 *gyr*A96 *rel*A1	Laboratory collection
Top10	F−*mcr*A Δ(*mrr-hsd*RMS-*mcr*BC) Φ80*lac*ZΔM15 Δ*lac*X74 *rec*A1 *ara*D139 Δ(*ara leu*) 7697 *gal*U *gal*K *rps*L (StrR) *end*A1 *nup*G	Laboratory collection
CC118(λpir)	Host strain for pKNG101 replication; Δ(*ara-leu*) *araD* Δ*lacX74 galE galK-phoA20 thi-1 rpsE rpoB argE* (Am) *recA1 Rf^r^ (*λ*pir)*	Laboratory collection
*P. aeruginosa*		
PAK	Wild-type prototroph	Laboratory collection
PAKΔ*retS*	*retS* deletion mutant	(15)
PAKΔ*retS*Δ*vgrG1a*	*vgrG1a* deletion mutant	(22)
PAKΔ*retS*Δ*vgrG1b*	*vgrG1b* deletion mutant	(22)
PAKΔ*retS*Δ*vgrG1c*	*vgrG1c* deletion mutant	(22)
PAKΔ*retS*Δ*vgrG1abc*	*vgrG1a*/*vgrG1b*/*vgrG1c* deletion mutant	(22)
PAKΔ*retS*Δ*tsei1–3*	*tse1tsi1*/ *tse2tsi2*/*tse3tsi3* deletion mutant	This work
PAKΔ*retS*v*vgrG1abc*Δ*tsei1–3*	*vgrG1abc*/*tsei1–3* deletion mutant	This work
PAKΔ*retS*Δ*vgrG1abc*Δ*tsei1–3::vgrG1a*	*vgrG1abc*/*tsei1–3* deletion mutant complemented in *trans* with *vgrG1a*	This work
PAKΔ*retS*Δ*vgrG1abc*Δ*tsei1–3::vgrG1b*	*vgrG1abc*/*tsei1–3* deletion mutant complemented in *trans* with *vgrG1b*	This work
PAKΔ*retS*Δ*vgrG1abc*Δ*tsei1–3::vgrG1c*	*vgrG1abc*/*tsei1–3* deletion mutant complemented in *trans* with *vgrG1c*	This work
PAKΔ*retS*Δ*tsei13*Δ*vgrG1abc*ΔH1::*vgrG1c*	*vgrG1abc*/*tsei1–3*/H1-T6SS deletion mutant complemented in *trans* with *vgrG1c*	This work
PAKΔ*retS*Δ*tsei13*Δ*vgrG1abc*Δ*rhsP1*::*vgrG1c*	*vgrG1abc*/*tsei1–3*/*rhsP1* deletion mutant complemented in *trans* with *vgrG1c*	This work
PAKΔ*retS*Δ*tsei1–3*Δ*vgrG1ab*	*vgrG1ab*/*tsei1–3* deletion mutant	This work
PAKΔ*retS*Δ*tsei13*Δ*vgrG1ab*Δ*rhsP1-CT*	*vgrG1ab*/*tsei1–3*/*rhsP1-CT* deletion mutant	
PAKΔ*retS*Δ*tsei1–3*Δ*vgrG1bc*	*vgrG1bc*/*tsei1–3* deletion mutant	This work
PAKΔ*retS*Δ*tsei13*Δ*vgrG1bc*Δ*PA0093*	*vgrG1bc*/*tsei1–3*/*PA0093* deletion mutant	This work
PAKΔ*retS*Δ*vgrG1a*Δ*PA0099*	*vgrG1a*/*vgrG1c*/*PA0099* deletion mutant	This work

**Plasmids**		
pRK2013	Tra^+^Mob^+^, K_m_^R^	Laboratory collection
pCR2.1	TA cloning vector, Ap^R^, K_m_^R^	Invitrogen
pCR-BluntII-TOPO	Blunt cloning vector, Zeo^R^, K_m_^R^	Invitrogen
pRL662-*gfp*	Gm^R^, broad host range vector derived from pBBR1MCS-2 expressing Gfp	Erh-Min Lai collection
pKNG101	Suicide vector, *sacB*, Str^R^	(39)
pKNG101-*vgrG1a*	pKNG101-*vgrG1a* mutator	(22)
pKNG101-*vgrG1b*	pKNG101-*vgrG1b* mutator	(22)
pKNG101-*vgrG1c*	pKNG101-*vgrG1c* mutator	(22)
pKNG101-*tsei1*	pKNG101-*tsei1* mutator	This work
pKNG101-*tsei2*	pKNG101-*tsei2* mutator	This work
pKNG101-*tsei3*	pKNG101-*tse3* mutator	This work
pKNG101-H1-T6SS	pKNG101-H1-T6SS mutator	(34)
pKNG101-*rhsP1*	pKNG101-*rhsP1* mutator	This work
pKNG101-*rhsP1-CT*	pKNG101-*rhsP1-CT* mutator	This work
pKNG101-*PA0093*	pKNG101-*PA0093* mutator	This work
pKNG101-*PA0099*	pKNG101-*PA0099* mutator	This work
Mini-CTX-1	Plasmid for the integration of genes into the *att* site of the *P. aeruginosa* chromosome, Tc^R^	Laboratory collection
Mini-CTX-*plac-vgrG1a*	Mini-CTX-1 encoding *vgrG1a* under a p*lac* promoter for integration into the *att* site of the *P. aeruginosa* chromosome for the purpose of complementation, Tc^R^	This work
Mini-CTX-*vgrG1b*	Mini-CTX-1 encoding *vgrG1b* under its native promoter for integration into the *att* site of the *P. aeruginosa* chromosome for the purpose of complementation, Tc^R^	This work
Mini-CTX-*vgrG1c*	Mini-CTX-1 encoding *vgrG1c* under its native promoter for integration into the *att* site of the *P. aeruginosa* chromosome for the purpose of complementation, Tc^R^	This work
pNDM220	Low copy number cloning vector containing *lacI^q^* and the LacI-regulated pA1/O4/O3	(36)
pBAD33	l-arabinose inducible, Cm^R^, p15A origin	(35)
pRhsP1-CT	pNDM220 expressing *rhsP1-CT*	This work
pRhsI1	pBAD33 expressing *rhsI1*	This work
pRhsP2-CT	pNDM220 expressing *rhsP2-CT*	This work
pRhsI2	pBAD33 expressing *rhsI2*	This work

*^a^* Ap^R^, ampicillin resistant; Str^R^, Streptomycin resistant; K_m_^R^, kanamycin resistant; Tc^R^, tetracycline resistant; Zeo^R^, zeocin-resistant.

##### DNA Manipulation

DNA purification was performed using the PureLink Genomic DNA minikit (Invitrogen). Isolation of plasmid DNA was carried out using the QIAprep spin miniprep kit (Qiagen). Restriction endonucleases were used according to the manufacturer's specifications (New England Biolabs or Roche Applied Science). Oligonucleotides used are shown in Table 2 and were purchased from Sigma. The genes or DNA fragments used for the construction of deletion mutants were amplified with KOD Hot Start DNA Polymerase (Novagen) as described by the manufacturer with the inclusion of Betaine (Sigma). Colony PCR was performed with standard Taq polymerase (New England Biolabs). DNA sequencing was performed by GATC Biotech.

**TABLE 2:** **Oligonucleotides used in this study**

	Oligonucleotide sequence*^a^*
**Deleted gene/oligonucleotide**	
*tsei1*	
Up5′	TGGACGTCACGGCTATAGGAA
Up3′	CCTGGGCAGCAGTTTCATGGGGCGGGTTCT
Down5′	ATGAAACTGCTGCCCAGGGCCAGTTGATTC
Down3′	AGGTCGTTGTTGCCTTTCAC
*tsei2*	
Up5′	ACCACTGGTCGACGAAGAGGTGGAC
Up3′	TCAGGATGCGTAGGACATGCGTGGACT
Down5′	ATGTCCTACGCATCCTGACGGCCCGTG
Down3′	CTTCAGCAGCAGCGCACCAGCTAC
*tsei3*	
Up5′	ACCAGACCCTGCAGCAATAC
Up3′	CTGCAACCTGGTCATCGGTCTTCCTCCTTG
Down5′	CCGATGACCAGGTTGCAGTGAGCGCGA
Down3′	CTGGAGGAAGCCGTATTCAG
*rhsP1*	
Up5′	GCTGAACGGCTTCGGCAGGTAGTC
Up3′	GATCTGCTTAATAGCCACGACACTCCT
Down5′	GTGGCTATTAAGCAGATCGTCAAATGC
Down3′	GTCGAGGGTACGCGAGGACATCAC
*PA0093*	
Up5′	GTTCCTCACCCGCATGCTCGAAG
Up3′	ATGGATGCGGGATCATGAATGACACCC
Down5′	CGCATCCATGCGTCGGTACTCGGGTCA
Down3′	GCCTTGATTCGCGAGCAACATTCCG
*PA0099*	
Up5′	AGTCGATTCCTACCTGACCG
Up3′	CTATGGACGGTTGGCCATCTAGTTCGC
Down5′	ATGGCCAACCGTCCATAGGAACTGAAC
Down3′	AGTAGAGCGGCAGGTTGG

**Amplified gene/oligonucleotide**	
*vgrG1a*	
5′	CGGGATCCTTCACACAGGAAACAGCTATGCAACTGACCCGC
3′	CGAGCTCTCAGCCCTTCGCCGGCGGCGGAAACAT
*vgrG1b*	
5′	GGATCCCTTTCGTGATGGCCTTCTG
3′	TGACTCCCTGGCTCTGCGCCGCTTCAGTTC
*vgrG1c*	
5′	GGATCCAGCATGATGTCCAGCAACAC
3′	AAGCTTCGCTCAACAGTTGATATCGAC

*^a^* Oligonucleotides are presented in the orientation 5′-3′.

DNA fragments encoding Rhs C-terminal putative toxin and putative immunity constructs were ordered as synthetic DNA constructs from GeneArt (Invitrogen). Constructs were designed using an 18-bp sequence including the Shine-Dalgarno sequence from pBAD (35) followed by an ATG start codon fused to the DNA sequence occurring directly after that encoding for the characteristic Rhs P*XXXX*DP*X*GL motif or the sequence of the predicted immunity gene followed by that encoding the V5his tag (from pET-DEST42 (Invitrogen)). The constructs encoding the putative C-terminal toxins and immunities were then subcloned from the GeneArt vectors into pNDM220 and pBAD33, respectively (36). DNA sequencing was performed by GATC Biotech.

##### Overexpression of Toxin/Immunity Pairs in E. coli

Growth curves were performed in 10-ml flasks. Briefly, overnight cultures of *E. coli* cells carrying plasmids encoding RhsP1-CT/RhsI1 or RhsP2-CT/RhsI2 were diluted in fresh LB media to an *A*_600_ of 0.1 with appropriate antibiotics. Cultures were incubated at 37 °C with agitation, and readings were taken hourly. Toxin/immunity expression was then induced with 1 mm isopropyl 1-thio-β-d-galactopyranoside or 0.02% arabinose, respectively.

##### Construction of P. aeruginosa Deletion Mutants

*P. aeruginosa* deletion mutants were constructed as described previously (37) using the suicide plasmid pKNG101 (38, 39). Briefly, to create PAKΔ*gene-of-interest* (*GOI*), 500-bp DNA fragments of the 5′ (up) and 3′ (down) ends of the target gene were obtained by PCR using PAK chromosomal DNA as the template with two pairs of oligonucleotides (Up5′/Up3′ and Down5′/Down3′) (Table 2). The fused up/down fragment was obtained by overlapping PCR, cloned into pCR-BluntII-TOPO (Invitrogen), sequence-confirmed, and subcloned into pKNG101 suicide vector. The plasmid pKNG-Δ*GOI* was maintained in the *E. coli* strain CC118λpir and mobilized into *P. aeruginosa* PAK using *E. coli* 1047 carrying the conjugative plasmid pRK2013 (40). Clones in which double recombination events occurred, resulting in the deletion of *GOI*, were selected on sucrose plates as previously described (37). Deletion of *GOI* was verified by PCR using external primers and Western blot analysis where appropriate.

##### Insertion of vgrG Genes at the P. aeruginosa Chromosomal att Site

For complementation of the triple *vgrG1abc* mutant by *vgrG1b* and *vgrG1c*, the DNA sequences including 300 bp upstream of the *vgrG1b* and *vgrG1c* genes were amplified and cloned into pCR-BluntII-TOPO. The genes were then excised and subcloned into Mini-CTX-1 (20) to construct Mini-CTX-*vgrG1b* and Mini-CTX-*vgrG1c.* As for *vgrG1a*, the gene was amplified and cloned under a *plac* promoter leading to the construct Mini-CTX-*plac-vgrG1a*. These plasmids were conjugated by tri-parental mating using pRK2013 into the appropriate *P. aeruginosa* strains, and the presence of the corresponding *vgrG* gene was confirmed by both PCR and Western blot analysis where appropriate.

##### Bacterial Competition Assay

Overnight cultures of *P. aeruginosa* strains were mixed individually with equivalent numbers of the prey *E. coli* DH5α pCR2.1 expressing β-galactosidase (34). After co-incubation on LB agar for 5 h at 37 °C, patches of bacteria were recovered and resuspended in Tryptone soy broth. Samples were serially diluted 0 to 10^−7^ and spotted in triplicate on LB plates containing 100 μg/ml 5-bromo-4-chloro-indolyl-β-d-galactopyranoside (X-gal) (Invitrogen). The level of blue color indicates survival of *lacZ-*positive *E. coli*. Alternatively, and for quantitative analysis of prey survival, *E. coli* DH5α containing pRL662*-gfp* was used as the prey, and spotting was performed on LB plates. The level of fluorescence indicates survival of *E. coli*. For quantification, the spots from the 10^−2^ dilution were resuspended in sterile PBS, diluted to 1:20, and pipetted into a 96-well, black/clear flat bottom plate (BD Bioscience, Falcon). Fluorescence emitted by the surviving prey cells was measured using a Fluostar Omega plate reader (BMG Labtech).

##### Subcellular Fractionation

*E. coli* strains producing recombinant proteins were collected by centrifugation and resuspended in 10 mm Tris, pH 8.0, supplemented with Complete Protease Inhibitor Mixture (Roche Applied Science) and 1 mm EDTA before cells were lysed by sonication (3 × 30 s, amplitude of 35%) using a Vibra-Cell ultrasonic processor (Sonics). Intact cells were removed by centrifugation at 4000 × *g* for 5 min at 4 °C. Soluble and membrane fractions were separated by centrifugation at 100,000 × *g* for 1 h at 4 °C. The membrane fraction was resuspended in 15 mm Tris, pH 7.4, supplemented with 1% Triton X-100 (Sigma) for 30 min at 4 °C. Soluble and insoluble membrane proteins were separated by centrifugation at 100,000 × *g* for 1 h at 4 °C. The proteins contained in the various fractions were analyzed by SDS-PAGE.

##### Separation of E. coli Membranes by Density Sucrose Gradient

*E. coli* strains producing recombinant proteins were harvested by centrifugation (2000 × *g*). The pellet of 250 *A*_600_ units equivalent of bacterial cells was resuspended in 1.5 ml of buffer A (10 mm Tris, pH 7.6, 10 μg/ml DNase (Sigma), Complete Protease Inhibitor Mixture (Roche Applied Science), and sucrose 20% (w/w) (Sigma)). The cells were passed through a French press cell disruptor (SIM-AMICO) at 1500 pressure units (p.s.i.) using a 3/8-inch-diameter piston (20K French pressure cell, Thermo). Unbroken cells and possible inclusion bodies were removed by two cycles of centrifugation at 4 °C for 15 min at 1600 × *g*. The resulting supernatant was centrifuged at 4 °C for 1 h at 125,000 × *g*. The collected crude membrane pellet was resuspended in 0.5 ml of buffer M (10 mm Tris, pH 7.4, complete protease inhibitor mixture (Roche Applied Science), and sucrose 20% (w/w)). The membrane fraction was then added on top of a discontinuous sucrose gradient consisting of 1.5-ml layers ranging from 60% (bottom) to 30% by 5% increments and separated according to the method described previously (41). Fractions of 500 μl were collected, and the protein content was analyzed by SDS-PAGE and Western blot.

##### Western Blot Analysis and SDS-PAGE

For SDS-PAGE analysis, cell extracts were loaded onto SDS-polyacrylamide gels, migrated, and transferred to a nitrocellulose membrane at 3 mA/cm^2^. After transfer, membranes were blocked overnight in blocking buffer (5% milk powder, 0.1% Tween 20 in Tris-buffered saline, pH 8.0). Polyclonal antibodies against VgrG1a were used at a dilution of 1:1000 (22). Monoclonal anti-V5 antibody (Invitrogen) was used at a dilution of 1:5000. Monoclonal antibodies were used against the β subunit of RNA polymerase (NeoClone) 1:1000. Antibodies against PhoE and SecA were kindly provided by Jan Tommassen (Utrecht University) and used at a dilution of 1:2000 and 1:1000, respectively. Secondary antibodies conjugated to horseradish peroxidase were used at a dilution of 1:5000. Western blots were developed using Super-Signal West Pico Chemiluminescent Substrate (Pierce) and visualized on a LAS3000 Fuji Imager.

##### Bioinformatic Analysis

DNA sequences were obtained from Pseudomonas Genome Database (42). Amino acid sequence searches were performed using SMART, Interscanpro, Pfam, BlastP, and PSI-BLAST software programs as implemented on the NCBI (National Center for Biotechnology Information) website. Secondary structure was predicted using PSI-PRED software program. Fold recognition was performed using Phyre2 (43) and I-TASSER (44). The TMHMM server was used to predict transmembrane helices in proteins.

## RESULTS

### 

#### 

##### A Broad Repertoire of Toxins Involved in H1-T6SS-dependent Killing

**FIGURE 1.**
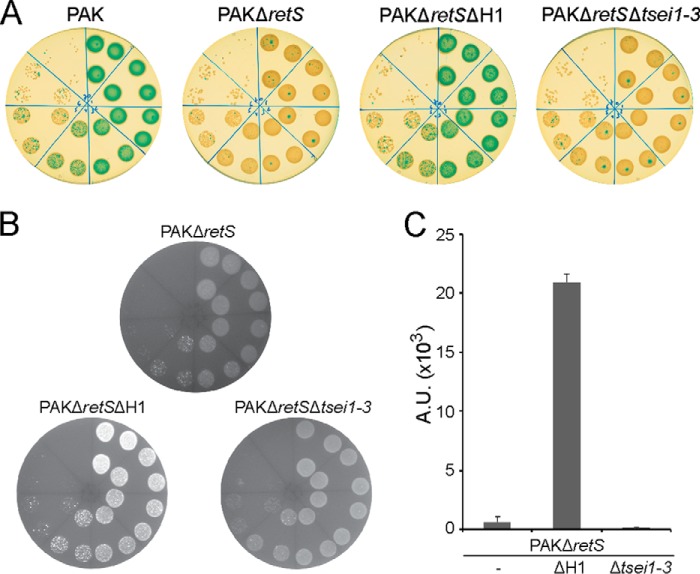
***P. aeruginosa* H1-T6SS-dependent bacterial killing involves more effectors than the Tse1–3 toxins.** *A*, cultures of *P. aeruginosa* strains PAK, PAKΔ*retS*, PAKΔ*retS*ΔH1, and PAKΔ*retS*Δ*tsei1–3* were mixed individually at a 1:1 ratio with *E. coli* DH5α prey cells expressing β-galactosidase (34). Recovered bacteria were diluted 0 to 10^−7^ and spotted on LB plates containing X-gal. The level of blue color indicates survival of *E. coli. B*, bacterial killing mediated by the strains PAKΔ*retS*, PAKΔ*retS* deleted for the H1-T6SS genes (PAKΔ*retS*ΔH1), or PAKΔ*retS* deleted for the *tse1–3* and *tsi1–3* genes (PAKΔ*retS*Δ*tsei1–3*) against DH5α expressing the green fluorescent protein. The level of fluorescence indicates survival of *E. coli. C*, quantification of the fluorescence shown in *B* as an indicator of bacterial killing. Bacterial spots were resuspended from the plates, and the residual fluorescence, reflecting *E. coli* survival, was measured and reported in arbitrary units (*A.U.*).

*P. aeruginosa* H1-T6SS secretes a set of three toxins, namely Tse1–3 (24), and the producing strains are protected from the activity of the Tse by cognate immunity proteins, Tsi1–3. The H1-T6SS and all three toxins/immunity pairs are up-regulated in a *retS* genetic background (23). The use of *E. coli* as target cells in an *in vitro* bacterial killing assay (34) shows a bactericidal activity mediated by a *P. aeruginosa retS* mutant but not by the parental PAK strain (Fig. 1). The deletion of the H1-T6SS cluster in the *retS* background (PAKΔ*retS*Δ*H1*) totally abrogates *E. coli* killing. By contrast, *E. coli* killing is still clearly observable upon simultaneous deletion of the *tse1–3* genes in the *retS* strain (PAKΔ*retS*Δ*tsei1–3*) (Fig. 1). This demonstrates the existence of additional uncharacterized H1-T6SS-dependent toxins.

##### VgrG Proteins Are Required for Toxin Delivery

**FIGURE 2.**
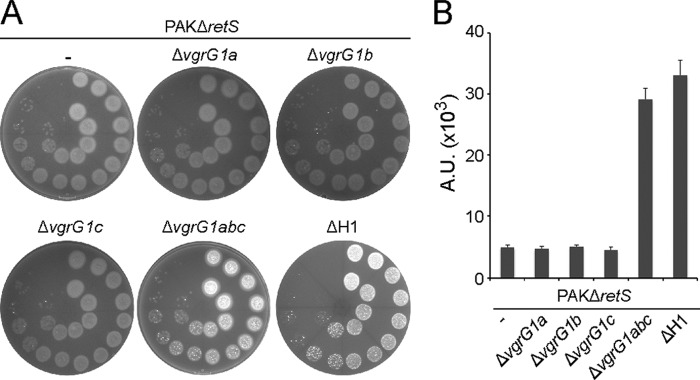
**VgrG1abc proteins are required for toxicity.** *A*, killing assay assessing the bactericidal activity of the *P. aeruginosa* strain PAKΔ*retS*, PAKΔ*retS* lacking either *vgrG1a*, *vgrG1b*, or *vgrG1c* (PAKΔ*retS*Δ*vgrG1a*, PAKΔ*retS*Δ*vgrG1b*, or PAKΔ*retS*Δ*vgrG1c*, respectively), lacking all three *vgrGs* (PAKΔ*retS*Δ*vgrG1abc*) and PAKΔ*retS* lacking the H1-T6SS cluster (PAKΔ*retS*ΔH1) against DH5α expressing GFP. The level of fluorescence indicates survival of *E. coli. B*, quantification of the bacterial killing mediated by the *vgrG* mutants. Bacterial spots were resuspended from *A*, and the relative fluorescence, reflecting *E. coli* survival, was measured and reported in arbitrary units (*A.U.*).

The *retS* regulatory pathway positively controls the expression of three *vgrG* genes, two of which are encoded within the H1-T6SS cluster, *vgrG1a* and *vgrG1b*, whereas the third, *vgrG1c*, is distally located (22). Individual *vgrG* gene deletion in the *retS* strain does not impact the ability of *P. aeruginosa* to kill *E. coli*; however, simultaneous deletion of all three *vgrG* genes totally abrogates killing (PAKΔ*retS*Δ*vgrG1abc*) (Fig. 2), just as observed with a H1-T6SS mutant. This observation suggests that if additional H1-T6SS-dependent toxins do exist, they require one of the VgrGs for transport and injection into target cells.

##### Genetic and Functional Linkage between vgrG1c and a rhs Gene

**FIGURE 3.**
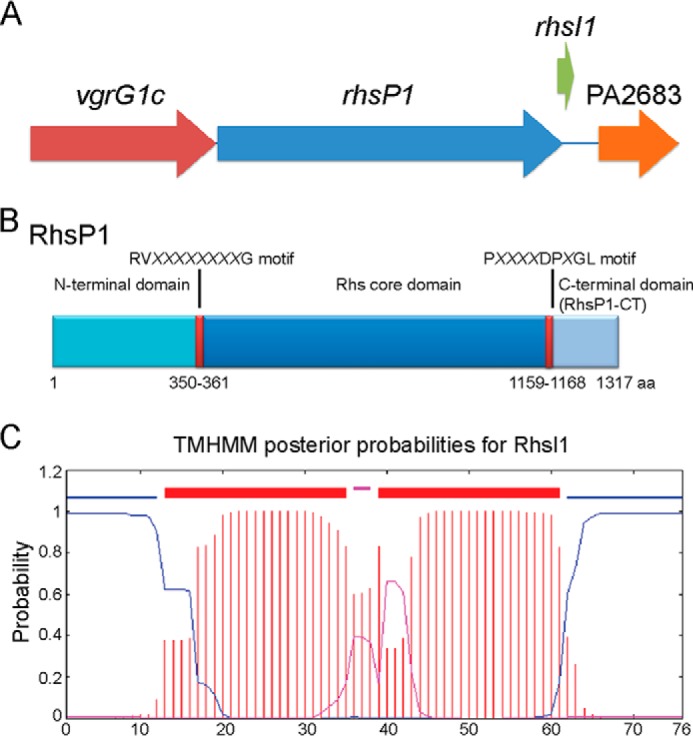
**A *rhs* gene is encoded downstream of *vgrG1c*.** *A*, genetic organization of the *vgrG1c* cluster showing *vgrG1c* (PA2685), *rhsP1* (PA2684), and the non-annotated immunity protein *rhsI1* and PA2683. *B*, schematic representation of RhsP1 displaying the N-terminal region, RV*XXXXXXXX*G motif, conserved Rhs core domain, P*XXXX*DP*X*GL motif, and the C-terminal domain (*RhsP1-CT*). *C*, TMHMM prediction of RhsI1 highlighting the two putative transmembrane domains.

**FIGURE 4.**
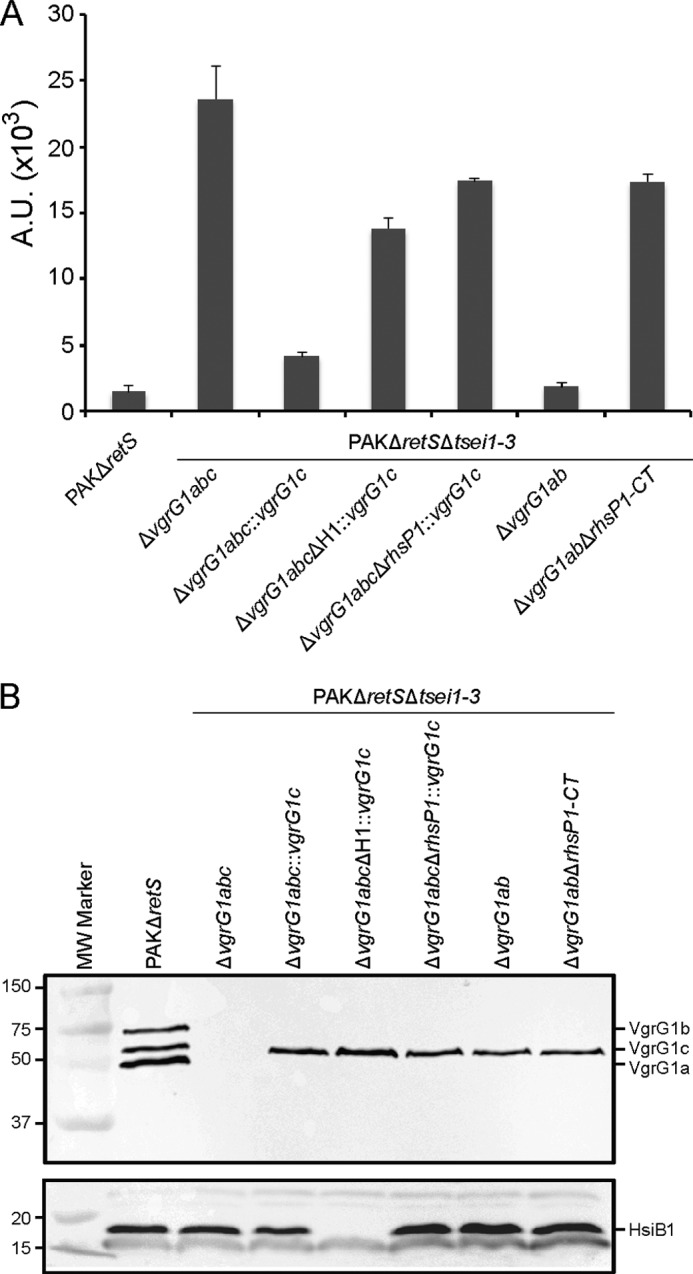
**RhsP1 is a H1-T6SS bacterial toxin that requires VgrG1c for killing.** The strains lacking all three Tse toxins (PAKΔ*retS*Δ*tsei1–3*) is used as the background strain with additional deletions (Δ) or insertion at the chromosomal *att* site (::) where indicated. *A*, quantification of bacterial killing by PAKΔ*retS*, PAKΔ*retS*Δ*tsei1–3*Δ*vgrG1abc*, PAKΔ*retS*Δ*tsei1–3*Δ*vgrG1abc*::*vgrG1c*, PAKΔ*retS*Δ*tsei1–3*Δ*vgrG1abc*ΔH1::*vgrG1c*, PAKΔ*retS*Δ*tsei1–3*Δ*vgrG1abc*Δ*rhsP1*::*vgrG1c*, PAKΔ*retS*Δ*tsei1–3*Δ*vgrG1ab*, and PAKΔ*retS*Δ*tsei1–3*Δ*vgrG1ab*Δ*rhsP1-CT* against DH5α expressing GFP. Bacterial spots were resuspended from plate killing assays, and the relative fluorescence was measured. The level of fluorescence indicates survival of *E. coli*. Complementation of *vgrG1c* in PAKΔ*retS*Δ*tsei1–3*Δ*vgrG1abc* restores *E. coli* killing. VgrG1c does not restore killing in a H1-T6SS or *rhsP1* mutant. PAKΔ*retS*Δ*tsei1–3*Δ*vgrG1ab*, which carries only the *vgrG1c* gene, readily kills *E. coli* (*second from the right*). This killing is abrogated if the region encoding the C-terminal domain of RhsP1 is deleted (*last bar*). *B*, the presence and absence of VgrG1c in the various genetic backgrounds was confirmed by Western blot analysis using anti-VgrG polyclonal antibodies (22). The presence and absence of H1-T6SS was confirmed by Western blot analysis using anti-HsiB1 polyclonal antibodies (20). *A.U.*, arbitrary units.

A plausible hypothesis is that if additional toxins need a VgrG protein for transport, the corresponding genes would be encoded in the vicinity of a *vgrG* gene. The *vgrG1c* gene is contiguous to a large gene (PA2684) that encodes a protein of the Rhs family (45), re-named RhsP1 (Fig. 3*A*). Previous reports indicated that Rhs proteins are involved in contact-dependent growth inhibition (CDI) and are thus potential bacterial toxins (46). A series of mutants have been engineered to assess the role of RhsP1 in bacterial killing and the possible connection with VgrG1c. To avoid any interference in our assay, these mutants were engineered in the PAKΔ*retS*Δ*tsei1-*3 strain. The deletion of the *vgrG1abc* genes yielded the PAKΔ*retS*Δ*tsei1–3*Δ*vgrG1abc* strain, lacking all *tse* and *vgrG* genes. As expected, this strain is completely innocuous for *E. coli* (Fig. 4*A*). The *vgrG1c* gene was then reintroduced at the *att* site on the chromosome of the PAKΔ*retS*Δ*tsei1–3*Δ*vgrG1abc* strain using a mini-CTX delivery system (PAKΔ*retS* Δ*tsei1–3*Δ*vgrG1abc*::*vgrG1c*). The presence/absence of VgrG1c in these genetic backgrounds was confirmed by Western blot analysis using anti-VgrG polyclonal antibodies (Fig. 4*B*). Remarkably, *E. coli* killing was readily restored upon complementation with the *vgrG1c* gene, which suggested that either VgrG1c has an intrinsic killing activity or that it is required for the transport of a toxin, which is not Tse1–3. It should be noted that the VgrG1c-dependent killing is H1-T6SS-dependent as deletion of this T6SS cluster in the PAKΔ*retS*Δ*tsei1–3*Δ*vgrG1abc*::*vgrG1c* strain abrogates *E. coli* killing (Fig. 4*A*). The lack of T6SS in this background was also assessed by Western blot using an antibody directed against the T6SS core component HsiB1 (Fig. 4*B*). We then tested whether RhsP1 could be associated with the VgrG1c-dependent killing. The *rhsP1* gene was deleted in the PAKΔ*retS*Δ*tsei1–3*Δ*vgrG1abc*::*vgrG1c* strain (PAKΔ*retS* Δ*tsei1–3*Δ*vgrG1abc*Δ*rhsP1*::*vgrG1c*), which yielded a strain innocuous for *E. coli*. We concluded that RhsP1 carries a bacterial toxin activity and that this protein is delivered to target cells in a VgrG1c-dependent manner.

##### The C Terminus of RhsP1 Is a Bacterial Toxin Domain

**FIGURE 5.**
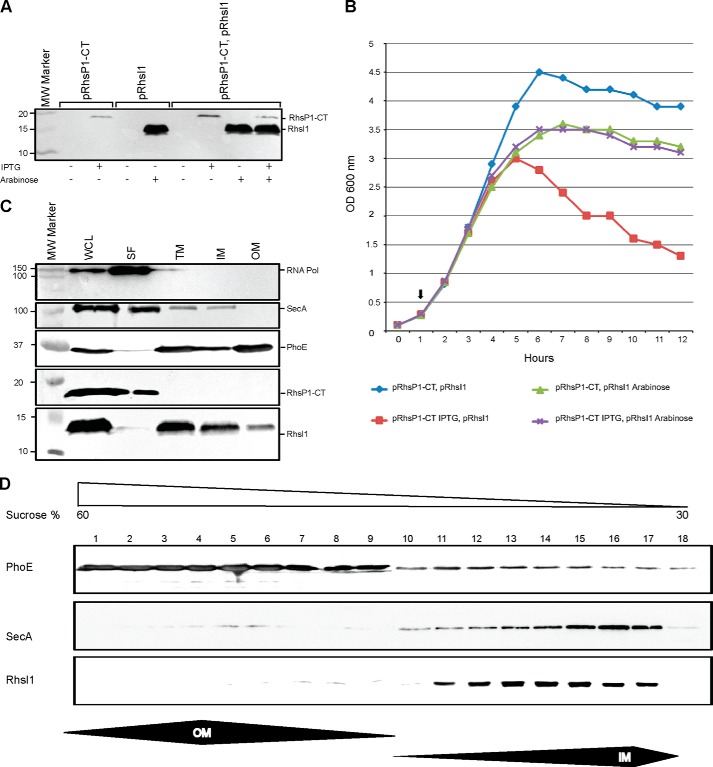
**RhsP1 and RhsI1 form a T6SS toxin/immunity pair.** *A*, Western blot analysis using an anti-V5 antibody demonstrating expression of RhsP1-CT and RhsI1. *B*, monitoring growth (*A*_600_) in LB medium of *E. coli* Top10 cells harboring pRhsP1-CT and pRhsI1 plasmids. Expression of RhsP1-CT and RhsI1 was induced at the time indicated (see the *arrow*) with either 1 mm isopropyl 1-thio-β-d-galactopyranoside (expression of the RhsP1-CT toxin) or 0.02% arabinose (expression of the RhsI1 immunity). Expression of RhsP1-CT compromised growth (*red curve*). Co-expression of RhsI1 rescue growth (*purple curve*). *C*, fractionation was performed to determine the subcellular localization of RhsP1 and RhsI1 using appropriate controls, *i.e.* RNA polymerase (*RNA Pol*, cytosolic), SecA (inner membrane-associated), and PhoE (outer membrane). Western blot analysis was performed using specific antibodies with the exception of the anti-V5 antibody, which was used to detect V5-tagged RhsP1 and RhsI1. RhsI1 is mostly located in the inner membrane fraction (*IM*), whereas RhsP1 is found in the soluble fraction (*SF*). *WCL*, whole cell lysate; *TM*, total membrane fraction; *OM*, outer membrane fraction. *D*, the crude membrane extract was separated on a density sucrose gradient by ultracentrifugation to determine the membrane localization of RhsI1 in *E. coli* as described previously (41). Collected fractions were analyzed by SDS-PAGE and Western blot using the antibodies described in *C*. The percentage of sucrose in the collected fractions (1–18) is shown on *top*, and the fractions containing either inner membrane (*IM*) or outer membrane (*OM*) proteins are delineated *underneath* the panels.

Rhs proteins, such as in *Dickeya dadantii*, are involved with CDI systems, and the toxic activity is carried in the C-terminal domain (Rhs-CT) (47). This Rhs-CT domain is clearly distinguishable and preceded by a P*XXXX*DP*X*GL motif. In case of RhsP1, the motif **P**TLAY**DP**T**GL** precedes a 149-aa long putative toxin domain (Fig. 3*B*). Furthermore in the CDI system, the toxin gene is usually organized in tandem with an immunity-encoding gene (*cdiI*). Although no obvious immunity gene is annotated directly downstream of PA2684 (*rhsP1*), a small *orf* overlapping with *rhsP1* could be predicted using ORF finder, which encodes a 76-aa-long protein (Fig. 3*A*). We named this putative gene *rhsI1* for *rhsP1 i*mmunity. To assess the functionality of RhsP1-CT/RhsI1 as a toxin/immunity couple, constructs encoding His_6_-V5-tagged versions were produced and cloned into compatible and independently inducible plasmids, pNDM220 and pBAD33 (35, 36). The production of both proteins was induced either by isopropyl 1-thio-β-d-galactopyranoside (pNDM220) or arabinose (pBAD33) and confirmed by Western blot analysis using anti-V5 antibodies (Fig. 5*A*). The impact of RhsP1-CT production on *E. coli* growth was then tested. Upon isopropyl 1-thio-β-d-galactopyranoside induction the toxin is produced, and growth inhibition could be observed by a drastic drop in the *A*_600_ of the culture 4 h post-induction (Fig. 5*B*). Remarkably, this drop in *A*_600_ is no longer observable when arabinose is also added to the culture, suggesting that RhsI1 is able to protect *E. coli* from the RhsP1-CT toxic effect (Fig. 5*B*).

The target for the toxic activity mediated by RhsP1 is unknown. RhsI1 is predicted to have two transmembrane domains, which likely results in the protein forming a hairpin loop in the cytoplasmic membrane (Fig. 3*C*). Subcellular localization experiments and Western blot analysis (Fig. 5*C*) show the presence of RhsI1 in the inner membrane. To further support this observation, the crude membrane fraction was loaded on a sucrose gradient (Fig. 5*D*). RhsI1 is mainly distributed in the fractions containing the inner membrane (from fractions 11 to 17 in Fig. 5*D*) along with the inner membrane-associated SecA protein. Instead, the outer membrane marker is more abundant in fractions 1–9 (Fig. 5*D*). RhsP1-CT appears mainly in the soluble fraction (Fig. 5*C*), suggesting it might be peripherally associated to the membrane upon interaction with the immunity protein.

Finally, we assessed the role of RhsP1-CT in the bacterial killing assay previously described. A strain deleted for *vgrG1a* and *vgrG1b* but in which the native *vgrG1c* gene is still present (PAKΔ*retS*Δ*tsei1–3*Δ*vgrG1ab*) shows significant toxicity against *E. coli* (Fig. 4*A*). In this background, deletion of the DNA region encoding the RhsP1-CT domain only (PAKΔ*retS*Δ*tsei1–3*Δ*vgrG1ab*Δ*rhsP1-CT*) abrogates *E. coli* killing ability (Fig. 4*A*), which confirmed the bactericidal activity conferred by the C terminus of RhsP1.

##### VgrG1a Mediates a PA0093-dependent Bacterial Killing

**FIGURE 6.**
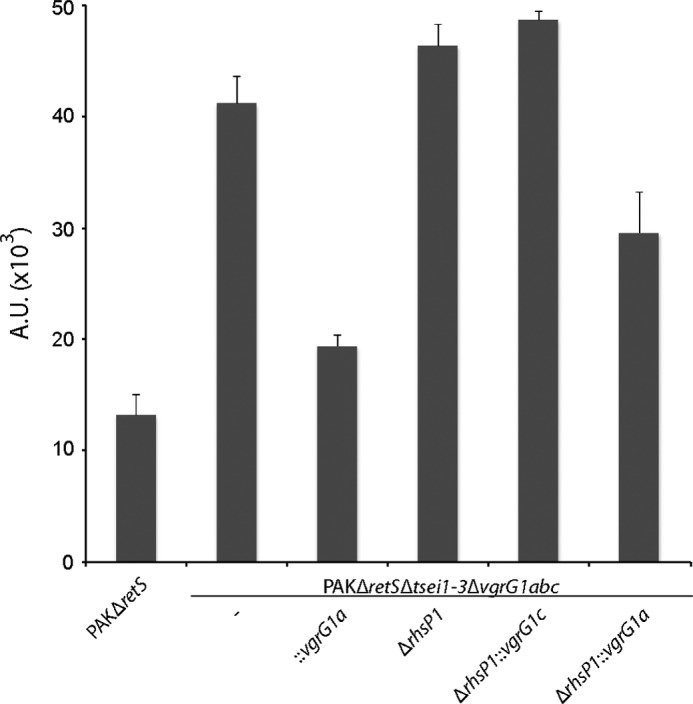
**Contribution of VgrG1a to the T6SS-mediated killing.** Quantification of bacterial killing by the *P. aeruginosa* strains PAKΔ*retS*, PAKΔ*retS*Δ*tsei1–3*Δ*vgrG1abc*, PAKΔ*retS*Δ*tsei1–3*Δ*vgrG1abc*::*vgrG1a*, PAKΔ*retS*Δ*tsei1–3*Δ*vgrG1abc*Δ*rhsP1*, PAKΔ*retS*Δ*tsei1–3*Δ*vgrG1abc*Δ*rhsP1*::*vgrG1c*, and PAKΔ*retS*Δ*tsei1-3*Δ*vgrG1abc*Δ*rhsP1*::*vgrG1a* against DH5α expressing GFP is shown. The level of fluorescence indicates survival of *E. coli*. Bacterial spots (similar to what is shown in Fig. 2*A*) were resuspended, and the relative fluorescence, reflecting *E. coli* survival, was measured and reported in arbitrary units (*A.U.*). Complementing the triple *vgrG* mutant with *vgrG1a* restores killing (compare the *second* and *third columns* starting from the *left*). VgrG1a-dependent killing could be observed in the absence of RhsP1, which is not the case for *vgrG1c* complementation (compare the *fifth* and *sixth columns*).

**FIGURE 7.**
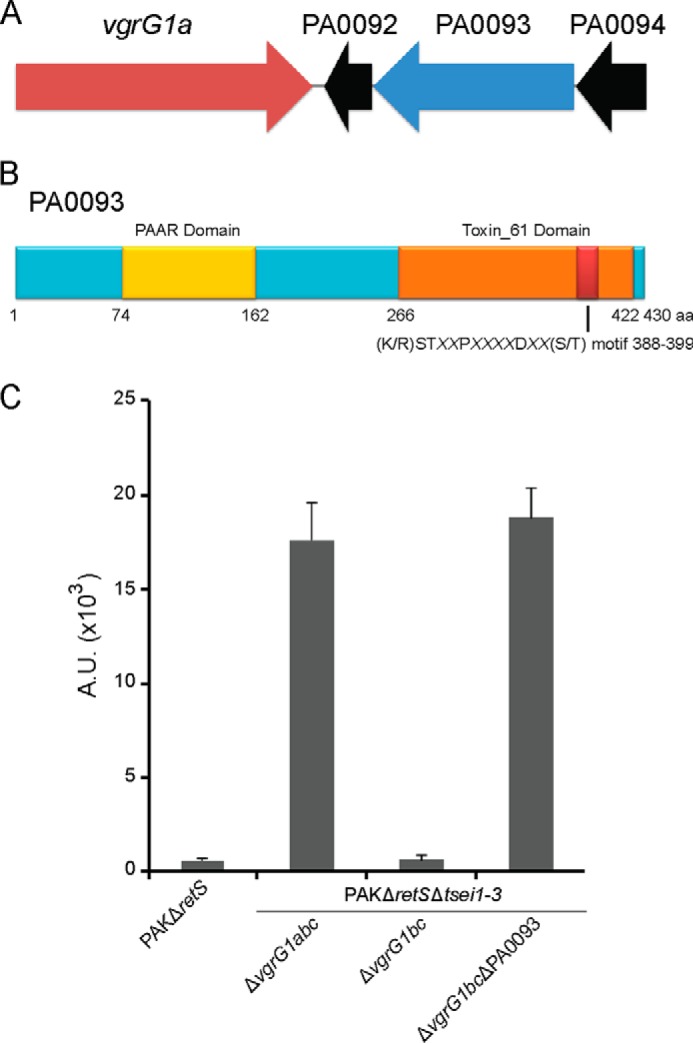
**PA0093 is a H1-T6SS effector protein that requires VgrG1a for killing.** *A*, genetic organization of the *vgrG1a* cluster *vgrG1a* (PA0091) in *red*, PA0093 encoding the putative toxin in *blue*, and genes with no clear function in *black. B*, A schematic illustration of the domain organization of PA0093 is shown. Indicated are the PAAR region, the predicted toxin domain (Toxin_61), and its associated conserved motif (K/R)ST*XX*P*XX*D*XX*(S/T) (**RST**AA**P**TD**D**FW**S**). *C*, quantification of bacterial killing using PAKΔ*retS*, PAKΔ*retS*Δ*tsei1–3*Δ*vgrG1abc*, PAKΔ*retS*Δ*tsei1–3*Δ*vgrG1bc*, and PAKΔ*retS*Δ*tsei1–3*Δ*vgrG1bc*Δ*PA0093* against DH5α expressing GFP. Bacterial spots (similar to what is shown in Fig. 2*A*) were resuspended, and the relative fluorescence, reflecting *E. coli* survival, was measured and reported in arbitrary units (*A.U.*). The presence of *vgrG1a* confers the ability to kill *E. coli*. The deletion of PA0093 abolishes this killing and highlights its role as a toxin dependent on VgrG1a.

The specific contribution of VgrG1c to the RhsP1-mediated toxicity led us to investigate whether other H1-T6SS-related VgrG proteins are linked to yet uncharacterized toxins. In the strain lacking all three *vgrG* genes, PAKΔ*retS*Δ*tsei1–3*Δ*vgrG1abc*, the *vgrG1a* gene was reintroduced at the *att* site on the chromosome (PAKΔ*retS*Δ*tsei1–3*Δ*vgrG1abc*::*vgrG1a*), as described previously. It was observed that killing is restored by the introduction of VgrG1a (Fig. 6), similar to what was previously observed with VgrG1c (Fig. 4*A*). In contrast to VgrG1c, the killing mediated by VgrG1a appears to be independent of RhsP1, as a strain lacking the *rhsP1* gene but expressing *vgrG1a* (PAKΔ*retS*Δ*tsei1–3*Δ*vgrG1abc*Δ*rhsP1*::*vgrG1a*) is able to kill *E. coli* prey cells, which is not the case upon complementation by *vgrG1c* (PAKΔ*retS*Δ*tsei1–3*Δ*vgrG1abc*Δ*rhsP1*::*vgrG1c*) (Fig. 6). This observation suggests a high degree of specificity for the VgrG proteins toward their cognate toxin partner (Fig. 6). Directly downstream of the *vgrG1a* gene lies a three-gene cluster (PA0092-PA0094). Among these, PA0093 encodes a 430-aa-long PAAR-containing protein (Fig. 7*A*). Some PAAR proteins (proline-alanine-alanine-arginine-repeat proteins) were proposed to be located at the tip of VgrG proteins (6). Furthermore, whereas the PAAR domain of PA0093 (aa 74–162) is found at the N terminus, the C-terminal region is predicted to carry a putative toxin domain (aa 266–422), named Toxin_61 (Fig. 7*B*), as defined using the comprehensive polymorphic toxin systems described by Zhang *et al.* (48). This particular domain displays an α-β fold and bears a conserved glutamine residue as well as a recognizable (K/R)ST*XX*P*XX*D*XX*(S/T) motif (**RST**AA**P**TD**D**FW**S**). In light of these observations, it is conceivable that PA0093 could use VgrG1a as a carrier. To assess this hypothesis, the gene encoded by PA0093 has been deleted in the strain PAKΔ*retS*Δ*tsei1–3*Δ*vgrG1bc* (PAKΔ*retS*Δ*tsei1–3*Δ*vgrG1bc*ΔPA0093), which is lacking *vgrG1b* and *vgrG1c* but still possesses the *vgrG1a* gene. Although bacterial killing is still potent when using a strain carrying only VgrG1a, the loss of PA0093 in this background totally abrogates the phenotype (Fig. 7*C*), thus confirming the functional relationship between VgrG1a and PA0093.

The toxic nature of PA0093 suggests that there may be an associated immunity. The two other genes in the PA0093 cluster, PA0092 and PA0094, encode proteins of 94 and 144 residues, respectively. Both proteins have neither predicted transmembrane domains (TMHMM) nor a signal peptide (SignalP 4.1), which suggests a cytosolic localization.

##### VgrG1b Contributes to Bacterial Killing

**FIGURE 8.**
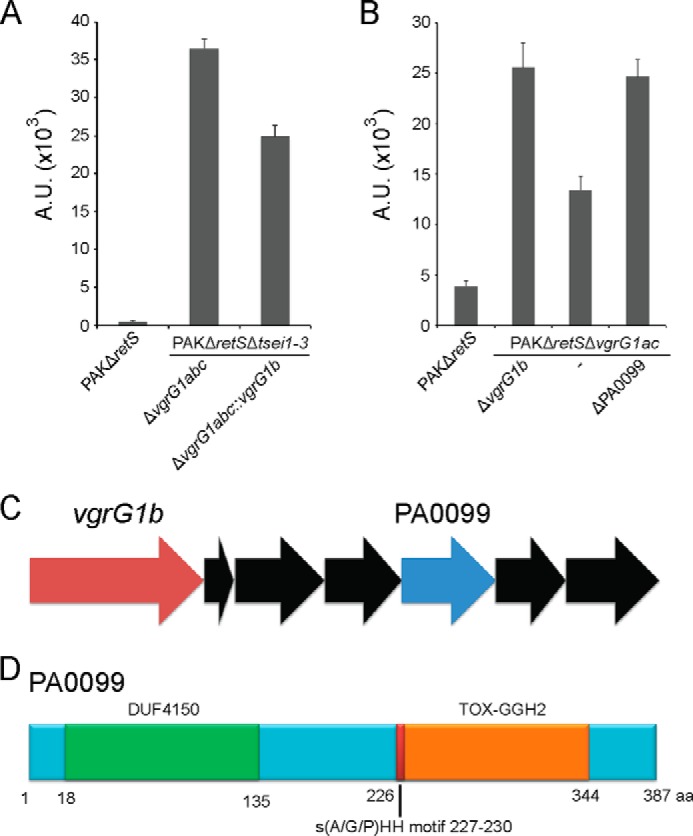
**PA0099 is an effector protein that requires VgrG1b for killing.** *A*, bacterial killing assay using the *P. aeruginosa* strains PAKΔ*retS*, PAKΔ*retS*Δ*tsei1–3*Δ*vgrG1abc* and PAKΔ*retS*Δ*tsei1–3*Δ*vgrG1abc*::*vgrG1b* against DH5α expressing GFP. Bacterial spots were resuspended from plates (as shown in Fig. 2*A*), and the relative fluorescence, reflecting *E. coli* survival, was measured and reported in arbitrary units (*A.U.*). The presence of VgrG1b contributes to the bacterial killing (*third lane*). *B*, quantification of bacterial killing assay using the *P. aeruginosa* strains PAKΔ*retS*, PAKΔ*retS*Δ*vgrG1abc*, PAKΔ*retS*Δ*vgrG1ac*, and PAKΔ*retS*Δ*vgrG1ac*Δ*PA0099* against DH5α expressing GFP. Quantification was performed as in *panel A*. Deletion of PA0099 (*fifth lane*) abrogates VgrG1b-dependent killing (*fourth lane*). *C*, genetic organization of the *vgrG1b* cluster highlighting *vgrG1b* (PA0095) in *red*, PA0099, the putative toxin, in *blue*, and genes with no clear function are colored *black. D*, schematic representation of PA0099 displaying the DUF4150 domain, the Tox-GHH2 domain, and the characteristic S(A/G/P)HH motif located within this domain.

According to our results, both VgrG1a and VgrG1c contribute to the delivery of specific effectors resulting in the killing of prey cells. To assess if such role could also be assigned to VgrG1b, the *vgrG1b* gene was reintroduced at the *att* site in a strain lacking all three *vgrG1abc* genes (PAKΔ*retS*Δ*tsei1–3*Δ*vgrG1abc*::*vgrG1b*). This strain clearly shows a partial recovery of killing ability (Fig. 8*A*). We hypothesized that the VgrG1b-dependent killing could be linked to another toxin encoded among the six genes of the cluster (PA0096-PA0101) lying downstream of *vgrG1b* (PA0095) (Fig. 8*C*). Careful analysis of the gene products from this cluster suggests a clear candidate, PA0099, that encodes a DUF4150-containing protein (aa 18–135) with a C-terminal putative toxin domain, TOX-GHH2 (aa 226–344), which is a GHH signature containing HNH/endonuclease VII superfamily nuclease toxin 2 with a characteristic S(A/G/P)HH signature motif (Fig. 8*D*). To verify this hypothesis, a double mutant lacking both the *vgrG1a* and *vgrG1c* genes (PAKΔ*retS*Δ*vgrG1ac*) and a triple mutant lacking *vgrG1ac* and *PA0099* were constructed. In the *vgrG1ac* mutant, *vgrG1b* is still present, and the strain is far more toxic to *E. coli* than the strain lacking all three *vgrG1abc* genes (Fig. 8*B*). However, upon introduction of the PA0099 deletion in the *vgrG1ac* mutant, the killing is totally lost. This observation suggests that the VgrG1b-dependent killing requires PA0099 and confirms the status of this protein as an anti-bacterial effector.

##### Another Rhs Toxin/Immunity Pair Is Encoded on the PA14 Genome

**FIGURE 9.**
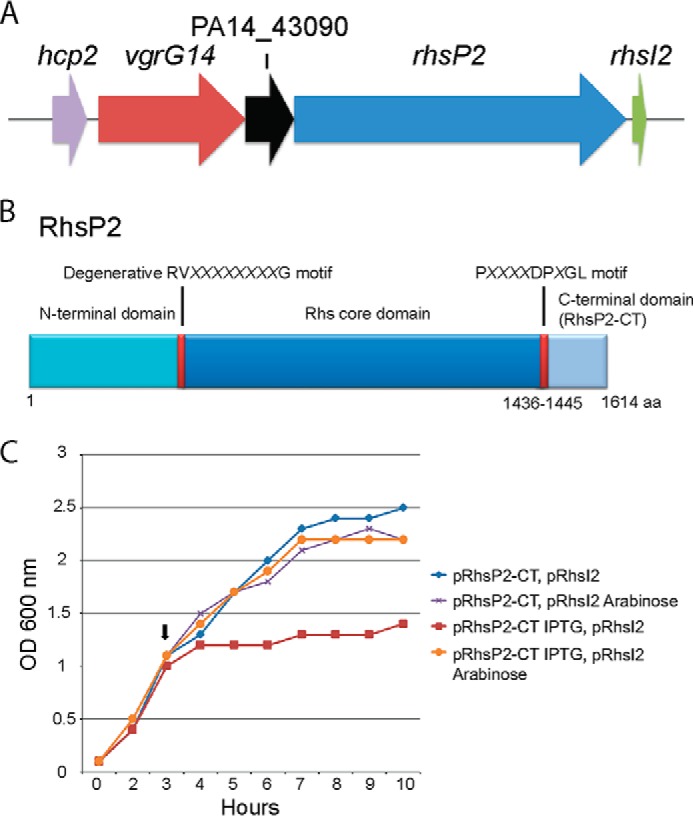
**RhsP2 and RhsI2 form a toxin/immunity pair.** *A*, genetic organization of the *vgrG14* cluster showing *hcp2* (PA14_43070), *vgrG14* (PA14_43080), a gene encoding a protein of unknown function PA14_43090, *rhsP2* (PA14_43100), and a small non-annotated ORF *rhsI2* encoding for a putative immunity protein. *B*, schematic representation of RhsP2 displaying the N-terminal region, RV*XXXXXXXX*G motif, conserved Rhs core domain, P*XXXX*DP*X*GL motif, and the C-terminal domain (*RhsP2-CT*). *C*, monitoring growth (*A*_600_) in LB medium of *E. coli* Top10 cells harboring pRhsP2-CT and pRhsI2. Cultures were induced at the time indicated (see the *arrow*) with either 1 mm isopropyl 1-thio-β-d-galactopyranoside (expression of the RhsP2-CT toxin) or 0.02% arabinose (expression of the RhsI2 immunity). Expression of RhsP2 compromised growth (*red curve*), whereas co-expression of RhsI2 rescues growth (*orange curve*).

The assortment of variable VgrG-associated toxins prompted us to investigate other related genomes, including the *P. aeruginosa* strain PA14. It has been previously shown that the *P. aeruginosa* PA14 strain carries additional genes in the vicinity of the H2-T6SS cluster that are absent in PAO1. This set of four genes (Fig. 9*A*) encodes a Hcp protein (Hcp2), a VgrG protein (VgrG14), a putative protein of unknown function (PA14_43090), and a Rhs element (PA14_43100) named RhsP2 (Fig. 9*B*) (49) and also carries a C-terminal domain (RhsP2-CT). The toxic activity observed for RhsP1-CT led us to further investigate the potential toxicity exerted by RhsP2-CT. Upon close inspection, a small ORF (435 bp) predicted to encode an associated putative immunity (RhsI2) was identified 9 bp downstream of the stop codon of *rhsP2* (Fig. 9*A*), which was not indicated in the original genome annotation. The genes encoding RhsP2-CT and RhsI2 were cloned separately into compatible plasmids as described for RhsP1. Overexpression of RhsP2-CT severely compromised growth of the producing strain (Fig. 9*C*), but co-expression of RhsI2 could rescue growth. This observation thus confirmed the existence of an additional toxin/immunity pair in selected strains of *P. aeruginosa*.

## DISCUSSION

The evolved *V. cholerae* VgrG1, displaying a C-terminal extension bearing an actin cross-linking domain (ACD), has been considered for a long time as the archetype substrate injected by the T6SS into eukaryotic cells (50–53). Since then only a few T6SS effectors have been shown to target eukaryotic cells (54). In more recent years the T6SS has been described as a potent weapon for bacterial warfare (2, 55). The discovery of three bacterial toxins delivered by the *P. aeruginosa* H1-T6SS has sparked this new concept, which is now widespread across Gram-negative bacterial species (56). Families of T6SS bacterial toxins have been studied in detail, such as the cell wall-degrading effectors (25). Two well characterized representatives, Tse1 and Tse3, are secreted by the H1-T6SS (24). Tse1 targets the peptide cross-links of the peptidoglycan, whereas Tse3 attacks the glycan backbone. Tse1 belongs to the family of type VI amidase effector (Tae), whereas Tse3 is a member of the T6SS glycoside hydrolase (Tge) (57). Other widespread toxins, such as the Type VI lipase effectors (Tle) have been shown to target cell membrane. In *P. aeruginosa*, Tle5 exhibits a phospholipase activity and has been suggested to be secreted by the H2-T6SS (58). Few other toxins, such as the bacteriostatic Tse2 toxin secreted by the H1-T6SS, have yet unknown function but are likely to exert toxicity within the cytoplasm (23). In all cases, each toxin is co-produced with an immunity protein, and the localization of the immunity matches the site of action of the toxin, *i.e.* cytoplasmic, periplasmic, or membranes. It is quite reasonable to propose that many more toxins could be identified and random approaches involving Tn-seq methodologies have uncovered yet unidentified T6SS immunity genes and subsequently pointed toward their cognate T6SS toxins (33).

In contrast to evolved VgrGs, the mechanism of T6SS-dependent transport of genuine bacterial T6SS toxins is open for discussion. A recent report proposed that Hcp acts as a chaperone for Tse2 (31). This observation suggests a delivery of this toxin to the target cell through the tubular structure of the T6SS (59–61). However, the *V. cholerae* VgrG3 is an evolved VgrG unrelated to Tae or Tge but is involved in the degradation of peptidoglycan in prey cells (32, 33). In this case, it could be proposed that the VgrG3 effector localizes at the tip of the T6SS tubular structure allowing it to reach the surface but would not travel within it. These various transport models have been discussed further in light of the characterization of PAAR components, which could be used as a linker between a non-evolved VgrG and a genuine T6SS toxin (6).

Here we characterized the contribution of three VgrG proteins, VgrG1abc (22), to the bacterial killing associated with the H1-T6SS of *P. aeruginosa* (34). The deletion of the three *vgrG* genes co-regulated with the H1-T6SS fully abrogated toxicity. Investigating the genetic environment of each one of the three *vgrG* genes revealed putative toxin-encoding genes. The gene located downstream of *vgrG1c* encodes RhsP1, and its C terminus, RhsP1-CT, contributes to bacterial killing. The toxin is neutralized by a putative immunity coded by a downstream gene, which was not annotated on the *P. aeruginosa* genome (62). This putative immunity protein has two predicted transmembrane domains and is located in the cytoplasmic membrane, suggesting that the target of RhsP1-CT is membrane-associated. The introduction of VgrG1c in a strain lacking the RhsP1-encoding gene does not restore killing, whereas introduction of VgrG1a in the same background results in bacterial killing. This suggests that in contrast to the Tse1–3-dependent killing, RhsP1 toxicity is specific to VgrG1c, which cannot be substituted with another similar protein. In fact, VgrG1a-dependent killing involves another toxin coded by the PA0093 gene located downstream of the *vgrG1a* gene. The C terminus of PA0093 is predicted (Pfam) to carry a putative toxin domain of unknown function (Toxin_61). Finally, VgrG1b independently contributes to the killing of *E. coli*. This is particularly interesting as VgrG1b secretion has been demonstrated to be H1-T6SS-independent (22). Bioinformatic analysis of the *vgrG1b* gene cluster (PA0096-PA0101) predicted PA0099 as a two-domain protein, with a C terminus belonging to the putative superfamily of nuclease toxins. Two Rhs proteins in *D. dadantii*, RhsA and RhsB, have been reported to carry a C terminus with a DNase activity (47). It was proposed that these proteins are involved in bacterial competition and requires adjacent *vgrG* genes to fulfill their function, although T6SS-dependent delivery involving other core T6SS components was not demonstrated.

Collectively, our data suggest that VgrG proteins could be considered as specific carriers for the delivery of cognate toxins. We identified three specific VgrG/toxin couples, namely VgrG1a/PA0093, VgrG1b/PA0099, and VgrG1c/RhsP1. It is likely that these combinations are not interchangeable, which is in marked contrast to what was observed for the Tse1–3 effectors. We can then speculate that each individual VgrG recognizes a cognate toxin and allow its delivery to a bacterial target. This mechanism could be related to the one exemplified by VgrG3 of *V. cholerae*, although in this case VgrG and the bacterial toxin are covalently linked (32, 33). In the case of VgrG1abc, the recognition is most likely indirect and may involve adaptor proteins such as PAAR proteins (6). Yet the putative PA0093 toxin associated with VgrG1a displays a PAAR domain at its N terminus, which may interact with VgrG1a. This configuration offers a combination in which the VgrG adaptor is not independent but connected to the toxin. The organization at the tip of the T6SS can thus be relatively diverse and would provide a modular platform for the positioning of a broad range of T6SS effectors. Nevertheless, VgrG proteins might still recognize more than one T6SS effector. For example, in *V. cholerae*, VgrG3 presents a built-in toxin at its C terminus but was shown to interact with another T6SS toxin, TseL, which displays a lipase activity.

The strict connection between VgrG and effectors could be a more general feature, and the delivery of these effectors can in some cases be independent of other core T6SS components. The role of VgrG1b in bacterial toxicity, although it has been shown previously to be H1-T6SS independent, supports this idea. The observation that RhsA and RhsB in *D. dadantii* are VgrG-dependent but have not been shown to be T6SS-dependent is also supportive of such a model. Finally, the existence of a large number of *vgrG* islands, which are not necessarily connected to T6SS core genes, also lends support to an original VgrG-dependent transport. For example, several additional *vgrG* gene clusters found in the *P. aeruginosa* genome present recognizable toxin-encoding genes. In the *P. aeruginosa* PA14 genome, PA14_69550 encodes VgrG5. A downstream gene, PA14_69520, encodes a protein of which the C terminus has a high similarity with the *E. coli* colicin 1a (63), as predicted using the Phyre software (43). The next gene, PA14_69510, encodes a protein with three predicted transmembrane domains, which is an ideal candidate to protect from the activity of this pore-forming protein. Another example is the *vgrG* cluster adjacent to the H2-T6SS cluster in PA14 and is lacking in PAO1 (49, 64, 65). In this case the *vgrG14* gene is clustered with a gene encoding RhsP2, whose C terminus displays a proven bacterial toxin activity.

A VgrG-dependent concept is an interesting development in T6SS research and could lead to distinguish an intermediate secretion strategy, more straightforward and relying exclusively on VgrG. This could be used for large-sized proteins and be similar to the CDI system that has been described in many instances and in which a combination of only two partners is involved (66). CdiA is a very large protein with a C terminus carrying the toxin activity and could thus be very comparable to the Rhs proteins. CdiA is transported by a single transporter of the TpsB/Omp85 family, CdiB. It is tempting to suggest that in some cases VgrG could act as a CdiB transporter and be the main facilitator in the secretion process, independently of T6SS core components. This remains an attractive hypothesis, but if true, it shall involve a different mechanism, which may explain many of the poorly understood aspects of the T6SS.

## Footnotes

4 The abbreviations used are:

T6SS: type VI secretion system
CDI: contact-dependent growth inhibition
aa: amino acids
CT: C-terminal
PAAR: proline-alanine-alanine-arginine-repeat protein.
